# Phenylephrine-induced recruitable preload from the venous side

**DOI:** 10.1007/s10877-018-0225-1

**Published:** 2018-11-26

**Authors:** Rita Jacobs, Stijn Lochy, Manu L. N. G. Malbrain

**Affiliations:** 1 Intensive Care Department, University Hospital Brussels (UZB), Laarbeeklaan 101, 1090 Jette, Belgium; 2 Cardiology Department, University Hospital Brussels (UZB), Jette, Belgium; 3 Faculty of Medicine and Pharmacy, Vrije Universiteit Brussel (VUB), Brussels, Belgium

## Shock is not always hypovolemia

During the perioperative period, patients often suffer from hemodynamic instability, especially after major abdominal surgery, due to several pathophysiological processes: anesthetics, hypovolemia, surgical procedure, mechanical ventilation, cardiac co-morbidities and patient positioning [1]. Over the last decades we have used aggressive fluid resuscitation as a cornerstone in the treatment of shock, however, with only little evidence to support this [2–4]. Much of our current traditions of fluid resuscitation comes from experience in treating the blue stage of spasmodic cholera, where giving large amounts of aqueous and saline injections saved lives [5]. Ever since, it is deeply incorporated in our culture that shock must always equal hypovolemia. Fluid administration to maintain or restore circulation hence became an integrated part in the care for patients undergoing surgery, but also in critically ill patients admitted to the ICU.

However, avoiding hypervolemia is mandatory (especially in situations of capillary leak and global increased permeability syndrome), since excessive fluid administration usually leads to edema, increased inflammation and permeability and compromised tissue healing [6–9]. To avoid excessive fluid administration leading to edema and fluid overload, while maintaining an adequate mean arterial pressure (MAP), vasopressors may be required. It has to be noted that the term fluid overload has recently been questioned and hyperhydration or intravascular versus extravascular hypervolemia may be more adequate terms [9].

## Venoconstriction as recruitable preload source

**Fig. 1:**
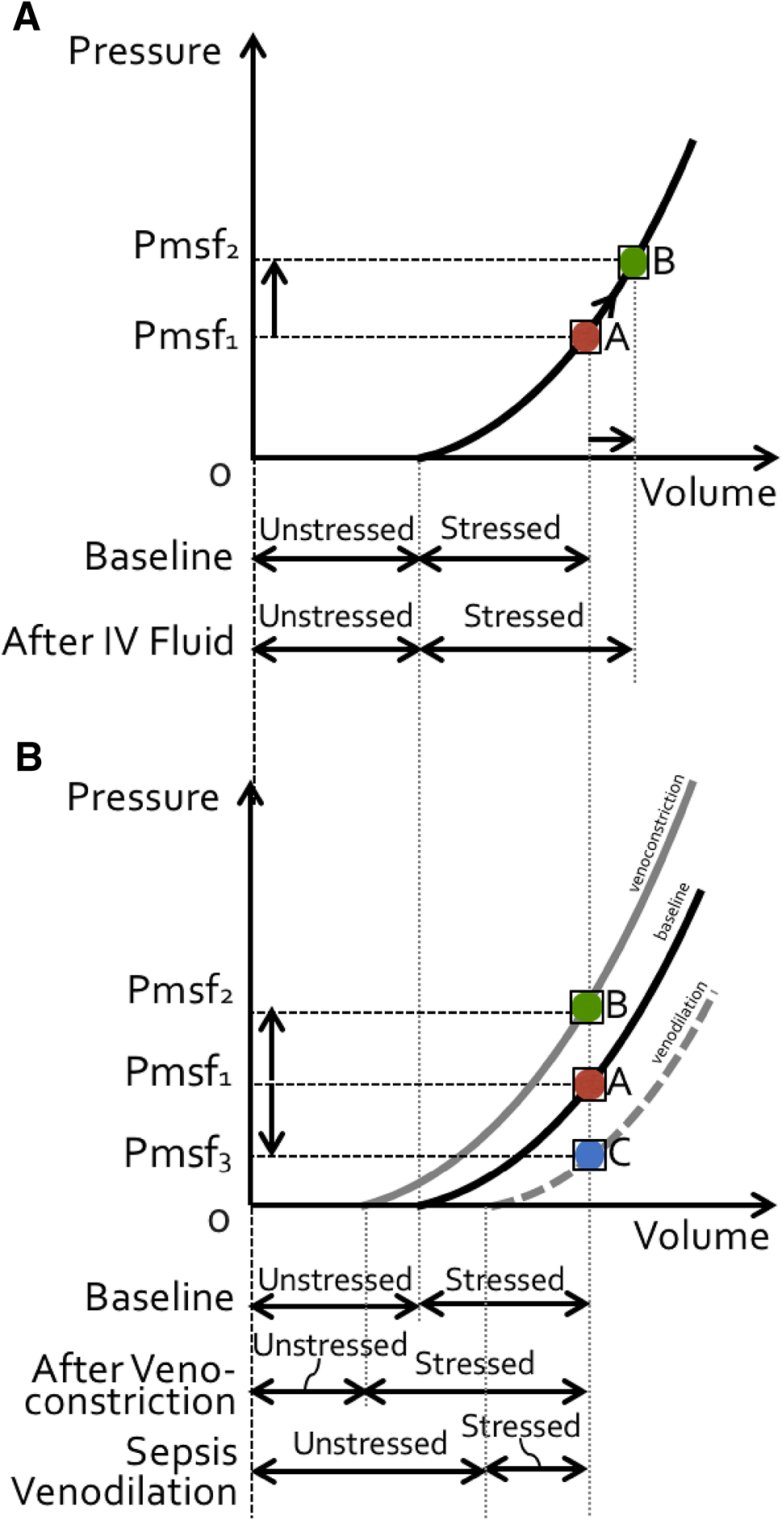
Effect of fluid loading and venoconstriction on volume. **a** Effect of volume loading on mean systemic filling pressure (*P*msf) and (un)stressed volume. Administration of a fluid bolus increases *P*msf (from *P*msf1 to *P*msf2, indicated respectively by position A (red dot) to B (green dot) on the pressure/volume curve). *Unstressed* volume remains constant while *stressed* volume increases. Total volume = unstressed + stressed increases, carrying a risk for fluid overload. See text for explanation. **b** Effect of venoconstriction and venodilation on mean systemic filling pressure (*P*msf) and (un)stressed volume. Venoconstriction increases *P*msf (from *P*msf1 to *P*msf2, indicated respectively by position A (red dot) to B (green dot) on the pressure/volume curve). *Unstressed* volume decreases while *stressed* volume increases. Total volume = unstressed + stressed remains constant, resulting in an auto-transfusion effect. Venodilation as seen in sepsis (vasoplegia) decreases *P*msf (from *P*msf1 to *P*msf3, indicated respectively by position A (red dot) to C (blue dot) on the pressure/volume curve). *Unstressed* volume increases while *stressed* volume decreases. Total volume = unstressed + stressed remains constant, resulting in an intravascular underfilling effect. See text for explanation

More than 70% of total blood volume (TBV) is located in the large veins, and increased vascular compliance (vasoplegia) like in sepsis or induced by anesthesia, can cause a substantial increase in TBV (with up to 80%) [10]. This increased venous compliance and increased TBV in early sepsis may represent a recruitable source of preload, as veins are very sensitive to low doses of vasopressor [11, 12]. These drugs directly convert unstressed blood volume to stressed blood volume while maintaining nearly normal venous elastance [11] (Fig. 1). Phenylephrine is widely used to treat intraoperative hypotension (via arterial and venous constriction). As a direct α-adrenergic receptor agonist, phenylephrine predominantly increases the systemic vascular resistance (SVR), systolic arterial pressure, MAP and left ventricular afterload, however its effects on cardiac output (CO) remain controversial [13]. The impact of phenylephrine on CO seems to be associated to preload dependency, where it generates most often an increase in CO through increased venous return. However, when the heart is preload independent, phenylephrine generally induces a decrease in CO, due to the increase in afterload [14–16].

Within this perspective, the recent publication by Wodack et al. elegantly described the effects of continuous administration of phenylephrine on the main determinants of CO [17]. The application of phenylephrine resulted in a significant increase in the volumetric preload variable global end-diastolic volume (GEDV), but not on the pressure-based preload variables central venous pressure (CVP) and an analog of mean systemic filling pressure (Pmsf). This effect is contributed to the effect of recruiting venous return from the venous capacitance pool, which then becomes available for the heart to generate stroke volume [18, 19]. This effect on preload volume was only observed after the first application of phenylephrine. However, a further increase in CO and cardiac contractility was observed, attributed to the positive inotropic effect of phenylephrine at higher dosages.

## All that glitters is not gold

Despite the interesting results, some methodological weaknesses should be considered.

First, as only eight pigs were studied, results cannot be translated into clinical practice.

Second, stroke volume variation (SVV) measured by arterial pulse contour analysis below 10% was used to determine a state of relative fluid unresponsiveness. However, although far better than static variables of LV preload, studies comparing pulse pressure variation (PPV) with SVV, found PPV to have a better predictive ability than the SVV [20].

Third, a further increase in CO, cardiac function index (CFI) and dPmax was attributed to a positive inotropic effect of phenylephrine. However, there was no correlation with echocardiographic measurements, the gold standard for assessment of LV contractility [21–23].

Fourth, another limitation is that phenylephrine changes vasomotor tone as well as venous capacitance. Vasopressors can significantly impair the ability of the dynamic preload indicators to predict fluid responsiveness, masking true intravascular volume deficit [24].

Fifth, the use of the mathematical modeling technique to determine Pmsf was based on MAP, CVP and CO. Any alteration in the measurement of these variables has an impact on the value of Pmsf [25]. Pmsf and CFI values were derived mathematically and are therefore, coupled with CO. Moreover, GEDV and CO values may also be mathematically coupled. Since CO increased with phenylephrine administration it is logic that GEDV increased following the formula:

$${\text{ITTV}}={\text{CO}} \times MTT,\
{\text{PTV}}={\text{CO}} \times DST\,{\text{and}}\,{\text{GEDV}}={\text{ITTV}}-{\text{PTV}}=\left( {{\text{MTT}}-{\text{DST}}} \right) \times {\text{CO}}$$

with MTT mean transit time, DST down slope time, ITTV intrathoracic thermal volume, PTV pulmonary thermal volume. Thus, when CO increases because of this mathematical coupling GEDV will also increase [26].

Sixth, there was no radiographic confirmation of the tip of the central venous catheter, which may have affected the accuracy of the CVP measurements.

Seventh, it would have been interesting to see the gradual effects and the amount of fluid loading needed in order to obtain the state of relative fluid unresponsiveness. In the present study, animals were already hemodynamically stabilized prior to start of the protocol.

## The case for mean systemic filling pressure

Pmsf is the blood pressure throughout the vascular system at zero flow and offers information on vascular compliance, volume responsiveness and it allows the calculation of (un)stressed volume. For determination of mean circulatory filling pressure two other bedside methods are available, either based on inspiratory hold-derived venous return curves (*P*msf hold), or on arterial and venous pressure equilibration (*P*msf arm) [27].

***P*msf hold** is based on the linear relation between CVP and venous return (*V*_R_):

$${V_{\text{R}}}=\left( {Pmsf - CVP} \right)/RVR$$

where RVR is the resistance to *V*_R_. Hereby, the CVP is raised by performing a series of end-inspiratory hold maneuvers and CO is measured in the last three seconds of the 12 s inspiratory hold. After 7–10 s a steady state occurs when *V*_R_ = CO. By plotting the CVP and CO values, a *V*_R_ curve is constructed and the zero-flow pressure (*P*msf) extrapolated [28, 29].

As *P*msf is defined as the steady-state blood pressure during no-flow conditions, the arm is used to estimate ***P*msf arm**. The upper arm is occluded to 50 mmHg above systolic blood pressure, using a rapid cuff inflator or a pneumatic tourniquet. Measurements of arterial and venous pressures through a radial artery catheter and a peripheral venous cannula in the forearm are performed. When these two pressures equalize, *P*msf arm values are obtained [28, 29].

## Venous capacitance

Most of the circulating TBV is located in the venous part, containing as stated previously approximately 70% of the body’s blood volume. Venous vascular beds consist of unstressed (70%) and stressed (30%) volume. Stressed volume represents blood volume in excess of the unstressed volume, and its volume in relation to venous motor tone defines the Pmsf [10].

Capacitance is the total contained volume that can be contained at a given pressure, and consists of stressed and unstressed volume. Stressed volume can be increased by decreasing vascular capacitance, which means recruiting unstressed volume into stressed volume. This is the equivalent of an auto-transfusion. Removal of sympathetic drive (e.g. during vasoplegia in sepsis or during sympathicolysis) can withdraw this equivalent amount of stressed volume and lead to a marked fall in mean circulating filling pressure (Fig. 1).

Under normal conditions, the body’s ability to rapidly vary unstressed volume is the primary means by which it alters venous return in response to changing metabolic demands [30].

The venous system contains α(1)-adrenergic receptors, and trough stimulation by phenylephrine the splanchnic capacitance vessels constrict [13]. However, phenylephrine also increases the venous resistance draining this region and the net effect on venous return is determined by how much volume is recruited versus how much the downstream resistance increased [13, 30].

## Conclusions

In conclusion, the large animal study performed by Wodack et al. on phenylephrine to treat perioperative hypotension is promising, since it does not only affect cardiac afterload, but also increases preload by shifting blood from the venous capacitance. This effect was confirmed by the observed increase in GEDV. Early application of vasopressors has the potential to reduce intravascular volume deficit by recruiting blood from the venous compartment (auto-transfusion effect), as well as to avoid the detrimental effects of fluid overload. Further studies are needed are to evaluate how these changes in management may affect outcomes.
